# An Accessible Multifunctional System to Support Safe and Independent Aging in Place: Iterative Development and Qualitative Analysis

**DOI:** 10.2196/72579

**Published:** 2025-10-16

**Authors:** Céline Madeleine Aldenhoven, Matthew Milton, Lisa D'Ambrosio, Chaiwoo Lee, Sophia Ashebir, Elisabeth André

**Affiliations:** 1 Software Engineering (Elite Graduate Program), Technical University of Munich, Ludwig Maximilian University, University of Augsburg, Munich and Augsburg, Germany; 2 AgeLab, Massachusetts Institute of Technology, 77 Massachusetts Ave, E40-275, Cambridge, MA, 02139, United States; 3 Department of Mechanical Engineering, Tufts University, Medford, MA, United States; 4 Chair for Human Centered Artificial Intelligence, Faculty of Applied Computer Science, University of Augsburg, Augsburg, Germany

**Keywords:** gerontology, aging in place, older adults, informal caregivers, mobile apps, self-determination theory, iterative design, qualitative text analysis

## Abstract

**Background:**

The global population of older adults is rapidly increasing, while the number of relative caregivers is declining. This creates a critical need for solutions that support caregiving and enable older adults to age in place while maintaining their independence. Many existing caregiving technologies focus on easing caregivers’ burdens through surveillance-based systems, which often prioritize caregivers’ needs over those of older adults. Such designs can unintentionally disempower older adults by devaluing their autonomy and decision-making capabilities.

**Objective:**

This study explored older adults’ and designers’ reactions to a technology-enabled system called LifeTomorrow that was built by centering older adults as the primary users. The system aims to support their autonomy to make informed choices and their desire for independent living, while balancing their social and functional needs with those of informal caregivers.

**Methods:**

A total of 37 participants, including older adults, caregivers, and designers, engaged in 2 iterative rounds of user studies to explore daily caregiving needs and technology usage. The system’s design and features were refined based on these insights. A qualitative thematic analysis was conducted using the framework of the self-determination theory to evaluate how the system fulfills the basic psychological needs of competence, autonomy, and relatedness.

**Results:**

The analysis underscored the system’s value to older adults and their caregivers, and its fulfillment of basic human psychological needs (competence, autonomy, and relatedness), consistent with the goals of supporting a high quality of life for older users and caregivers. We found that relatedness is fostered through features enabling remote connection and communication, such as chat functions and shared health data. Autonomy is supported by empowering older adults to manage their health information, make informed choices about data sharing, and benefit from safety features like fall detection and emergency calls. Competence is enhanced through accessible design elements, including intuitive navigation, high-contrast visuals, and multigenerational usability. These features allow older adults and caregivers to confidently engage with the system and are targeted at improving their overall quality of life.

**Conclusions:**

Through evaluation of the LifeTomorrow system, this study suggests possibilities for using a holistic, inclusive solution to support safe and independent aging in place and prioritizing the autonomy and empowerment of older adults while addressing caregivers’ needs for support and connection. By centering older adults as active participants rather than passive recipients of care, the system exemplifies a shift toward equitable, user-centered technology in caregiving. Future research should investigate the long-term impacts of such systems on aging-in-place outcomes.

## Introduction

### Background and Motivation

In the United States, 77% of adults aged 50 years or older desire to age in place, living independently in their homes for as long as possible [1]. However, an estimated 70% of people in the United States aged 65 years or older will require ongoing care and support at some point [2], often in their homes. This care is most often provided by informal or family caregivers [3]. Caregivers may assist their care recipients with a range of different tasks, from helping with instrumental activities of daily living (eg, transportation and managing finances) to providing direct health care support [4]. As a result of their caregiving, caregivers often experience negative impacts on their own physical and emotional well-being (eg, [5-7]). As the global population of older adults and their associated care needs rapidly increase, the demand for caregiving is also projected to grow [8-10]. Between the increasing demand for caregiving and the burden of caregiving on caregivers, there is an urgent need for solutions that support caregiving while respecting older adults’ desires for autonomy and independence [11].

Technology offers significant potential to address these challenges, yet many existing systems are designed primarily with caregivers’ needs in mind. These systems often prioritize reducing caregiving burden through surveillance and monitoring technologies, implicitly disempowering older adults by framing them as passive recipients of care [12-15]. Moreover, such solutions are frequently intrusive and expensive, and fail to center older adults as autonomous individuals capable of making decisions about their own care.

This study takes an alternative approach, examining user responses to a technology-enabled system designed with older adults’ needs at its core. We build upon previous knowledge that many older adults are open to adopting mobile or wearable health technologies, especially to share data with their health care providers [16]. The LifeTomorrow system aims to empower healthy older adults to live safely and independently in their homes while supporting informal caregivers. By fostering communication, enabling selective sharing of health information, and providing caregivers with access to resources, the system was built to try to address the needs of both older adults and caregivers without relying on direct surveillance or intrusive monitoring.

Through 2 rounds of user studies, we explored the reactions of older adults, informal caregivers, and designers to the LifeTomorrow system. Using self-determination theory [17,18] as an analytical framework, we examined the extent to which the LifeTomorrow system fosters competence, autonomy, and relatedness, which are fundamental psychological needs critical to maintaining a high quality of life for older adults and their informal caregivers.

### Prior Work

#### Technologies for Aging in Place

Aging in place refers to living within one’s community with some level of independence for as long as possible, rather than entering residential care in later life [19]. To do so safely requires affordable, unobtrusive, and easy-to-use solutions for older adults. Health-related innovations in human-computer interaction (HCI) research have great potential to enhance older adults’ access to health care resources while supporting long-term independence at home [20]. By developing intuitive, personalized, and accessible technologies, HCI can bridge gaps in health care delivery, promote self-management, and facilitate aging in place. For instance, voice assistants tailored for older adults can assist with health self-management tasks, such as debriefing doctor’s visit notes and generating medication reminders, enhancing adherence and understanding. Mobile health apps designed with input from older adults and their support networks can facilitate communication with caregivers and provide timely health information, promoting engagement and autonomy [21].

Despite the common stereotype of older adults not using technology, as of 2021, 61% of older adults in the United States had smartphones [22], and 70% of older adults who are online use technology daily [23]. Older adults’ intentions to use smart home technologies, which offer the potential to support greater in-home care and foster independence, are influenced by factors such as ease of use, accessibility, privacy, security, and cost [24]. Despite older adults’ openness to health-monitoring technologies, barriers like steep learning curves and usability concerns remain significant challenges [25-28]. Designing technologies that emphasize simplicity, intuitive interfaces, and comfort is essential to promote adoption. Additionally, systems should accommodate support needs, such as setup assistance and ongoing technical help [29].

Several smart home care systems use cameras or voice recognition [30-36], but these raise privacy and security concerns. For example, Trajkova et al [37] found that older adults often limit or abandon voice assistants due to challenges with finding valuable uses, beliefs, and shared spaces.

A number of different systems also depend on more complicated architectures and physical components. For example, numerous studies have explored the use of a large number of sensors to support aging in place [38-41] and monitor daily activity phases [42]. While these systems may provide caregivers with valuable insights, they typically emphasize the need for caregivers to know where their care recipient is and what they are doing, overlooking older adults’ autonomy. Moreover, many solutions rely on large sensor networks or machine learning systems that require substantial infrastructure or assume routine behavior patterns, which may not suit more active older adults [43,44].

#### Technologies to Support Caregivers

Remote caregiving technologies primarily aim to reduce caregivers’ burdens by providing tools to monitor health, safety, and well-being from a distance [45]. Wearable devices and in-home monitoring systems can share vital health and safety information with caregivers and health care providers [46,47]. Additionally, internet-based interventions, such as virtual support groups and educational resources, have been shown to improve caregiver well-being by helping to manage caregivers’ depression, reduce stress and anxiety, improve self-efficacy, and increase confidence [47-50].

Despite the benefits they may offer, much of the existing work on caregiving technologies focuses on narrower, piecemeal solutions addressing specific issues such as fall detection [51], activity monitoring [52], or communication and social isolation [49,53]. Systems often lack the capacity to address broader caregiving challenges, such as caregivers’ mental health or access to resources. One exception is the SOS caregiver service by Graffigna et al [54], which offers a promising model for reducing caregiver isolation and providing access to knowledge, but most solutions fail to provide the same level of holistic support [55].

#### Supported Needs and Limitations

Because many current systems are designed around caregivers’ needs and desires for information about the status of the care recipient, older adults may feel surveilled or disempowered and like their autonomy has been undermined. Privacy, stigma, and usability challenges further complicate adoption [26,56,57].

While collecting and sharing personal health data from older adults with caregivers may enable more customized care [45,58], concerns remain about reduced face-to-face interaction. It is vital to enhance, not replace, personal contact to prevent loneliness and social isolation [19,58].

For technological systems to achieve widespread adoption, they must also be cost-effective. Projects like the smart home system by Raad et al [39] and the SIMBAD project [59] are efforts to address this by using low-cost remote monitoring systems and fall detection sensors, respectively. Similarly, the survey by Visutsak et al [60] highlights the preference for wearable devices like smartwatches to support independent living within constrained budgets. While these examples provide critical functionality at a lower cost, they still largely focus on reactive or deficit-oriented designs, addressing specific concerns such as falls or emergencies.

In this research, we evaluated user responses to a system that follows a holistic design centering on the needs of older adults [47]. The approach to the system design emphasized empowering older adults as autonomous individuals while supporting informal caregivers with practical tools and resources. Unlike many existing systems, this system aims to avoid intrusive surveillance, respect privacy, and be cost-effective in order to improve accessibility and adoption. The research here explores users’ and designers’ reactions to 2 iterations of the prototype system designed to center and support human agency.

#### Analysis Framework: Self-Determination Theory

Self-determination theory was proposed by Deci and Ryan [61] in 1985, with a focus on an individual’s ability to self-assure, make decisions, and think independently. It suggests that individuals need to meet their basic psychological needs, which include maintaining social connections (relatedness), feeling competent (competence), and self-regulating feelings and behaviors (autonomy) [17]. Satisfying these needs leads to a positive emotion and a sense of subjective well-being in a person’s growth and development [17,61].

Self-determination theory is crucial in understanding HCI and technology use. Studies on gaming often use it to understand players’ demand fulfillment, intrinsic drive, and addiction-like sensations [62], but in recent years, it has also been applied to people’s interactions with whole-body control interfaces [63], artificial intelligence systems [64,65], and robots [66,67]. For example, Schwaninger et al [68] identified various types of relatedness, such as interpersonal and institutional, in people’s usage of remote health monitoring technology. Zhao et al [18] applied self-determination theory to explore how using digital technologies for meaningful activities can support or undermine older adults’ needs for autonomy, competence, and relatedness. Yang et al [65] leveraged self-determination theory to explore how users’ competence, autonomy, and relatedness needs could be supported or undermined in experiences with conversational agents.

Competence, relatedness, and autonomy are also predictive and reliable mediators of motivation, engagement, and well-being [17,69]. The design of interactive applications has been effectively guided by self-determination theory to produce positive experiences [18,65,70,71]. In this work, we used self-determination theory to analyze how our proposed system contributes to fulfilling 3 basic human psychological needs (competence, autonomy, and relatedness) [17], consistent with the goals of supporting agency for older users and a high quality of life for them and their informal caregivers.

## Methods

### Study Procedure

**Figure 1.**
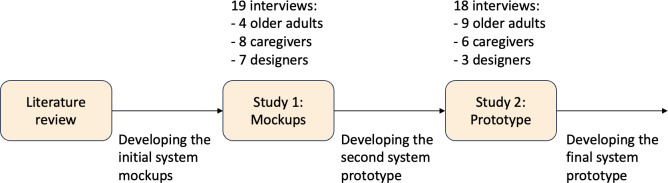
Research process and system iterations.

Two rounds of user studies were conducted iteratively to improve the system design. Semistructured interviews facilitated participants’ sharing of personal experiences and allowed tailored follow-up questions [72]. We built on an approach for analyzing think-aloud usability test sessions [73], which allowed people to engage with and react to the system design and prompts naturally and to respond using their own thoughts and words. The research process is outlined in Figure 1, and example questions from the interviews can be found in Multimedia Appendix 1.

The first round of user studies for mockup evaluation was conducted online via Zoom (Zoom Communications, Inc), enabling broad participant inclusion regardless of location. After providing informed consent and demographic information, participants received an overview of the LifeTomorrow system. Older adults and informal caregivers reviewed mockups specific to their roles, while designers evaluated the full system. Participants provided feedback on the design and usability of each screen, discussed privacy concerns, and shared overall impressions. Concluding questions explored their likelihood of using or recommending the system.

The second round of user studies for prototype evaluation was conducted in person at the Massachusetts Institute of Technology (MIT) campus, enabling hands-on interaction with the implemented prototype. Participants provided written informed consent and demographic information on large-print forms. Older adults and informal caregivers tested their respective prototype apps on devices (iPad and Apple Watch for older adults; iPhone for informal caregivers) by completing tasks in the system with only a few sentences of introduction to their purpose. Tasks included finding specific information, sharing data, and navigating features, with task order randomized. Designers reviewed the complete system. Participants provided feedback on individual screens, privacy concerns, and system updates since round 1. Final questions addressed usability and willingness to recommend the system.

### Study Stimulus: Overview of the LifeTomorrow System

The LifeTomorrow system is a multifunctional app designed to support independent living for older adults and their informal caregivers [74]. The system prioritizes support for older adults’ independence [1]. Moving away from “elderly surveillance systems” [13], it allows older adults to manage their health data independently, monitor their personal data sharing, and grant or revoke access to their data at any time [12-14].

System functionalities include fall detection and communication, monitoring and display of vital health signs, emergency communication, and environmental safety (fire, smoke, and carbon monoxide detection), with an overall design flexible enough to enable the addition of future sensing and monitoring devices.

For older adults, an Apple Watch with automatic fall detection and emergency call capabilities is used, removing the need for specialized emergency call systems. The smartwatch’s cellular connectivity and health tracking ability (eg, step counts, heart rate, and blood oxygen monitoring) eliminate the need for a smartphone or separate tracking device. The system includes a network of sensors in the older adult’s home, including smart carbon monoxide detectors and a stove sensor. Collectively, the system provides a safer home environment, with capabilities to detect and notify events such as falls and fire hazards [75,76], which disproportionately have detrimental impacts on older adults. The system is further designed with flexibility to add other smart sensors, such as a door contact sensor, using the Matter framework [77,78], which allows interconnectivity between smart sensors from different manufacturers for different platforms. Overall, the system leverages products that are widely available and used, in order to make the system more accessible for economically less secure older adults [74,79] compared to large sensor networks [43,44].

**Figure 2.**
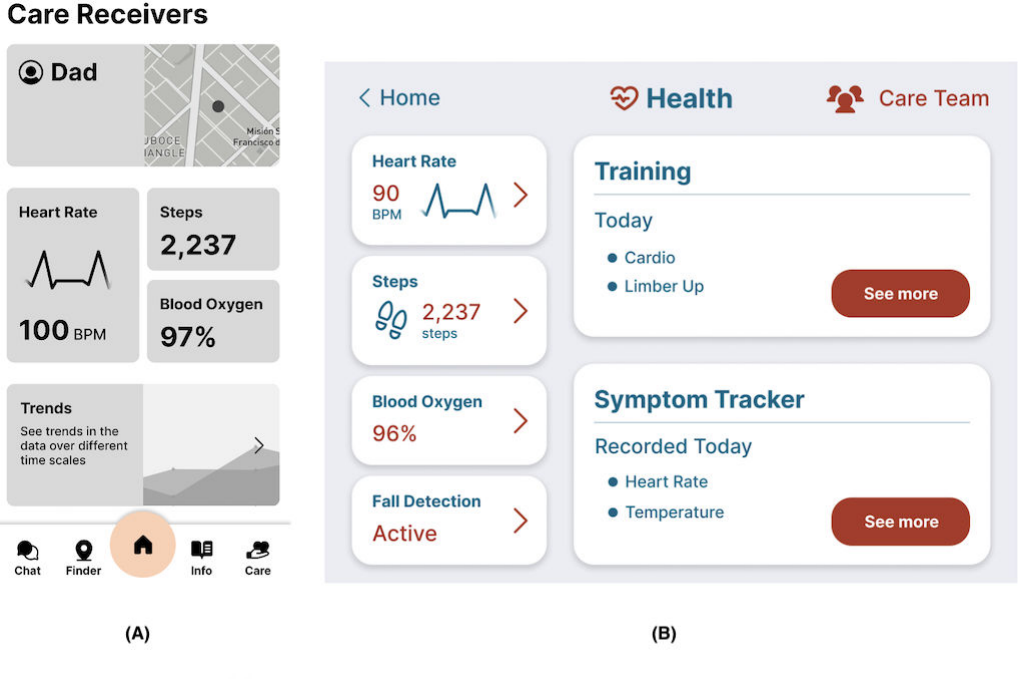
Initial system used for round 1: (A) Caregiver’s LifeTomorrow app; (B) Older adult’s LifeTomorrow app.

The data from the Apple Watch can be displayed on an iPad using Apple’s secure health data application programming interface, HealthKit [80], on iPad OS 17 Developer Beta, along with the data from the sensors. Inspired by previous research [81-83], we focused on an accessible design for older adults, including easy and intuitive navigation through the app. The app’s interface is designed with an accessible color palette and high contrast, ensuring a better user experience. The 2 rounds of user studies were conducted to improve and redesign user interface (UI) visualizations and interactions as needed (Figure 1). Example screens after each round of user studies illustrating the system’s evolution can be found in Figures2  3.

**Figure 3.**
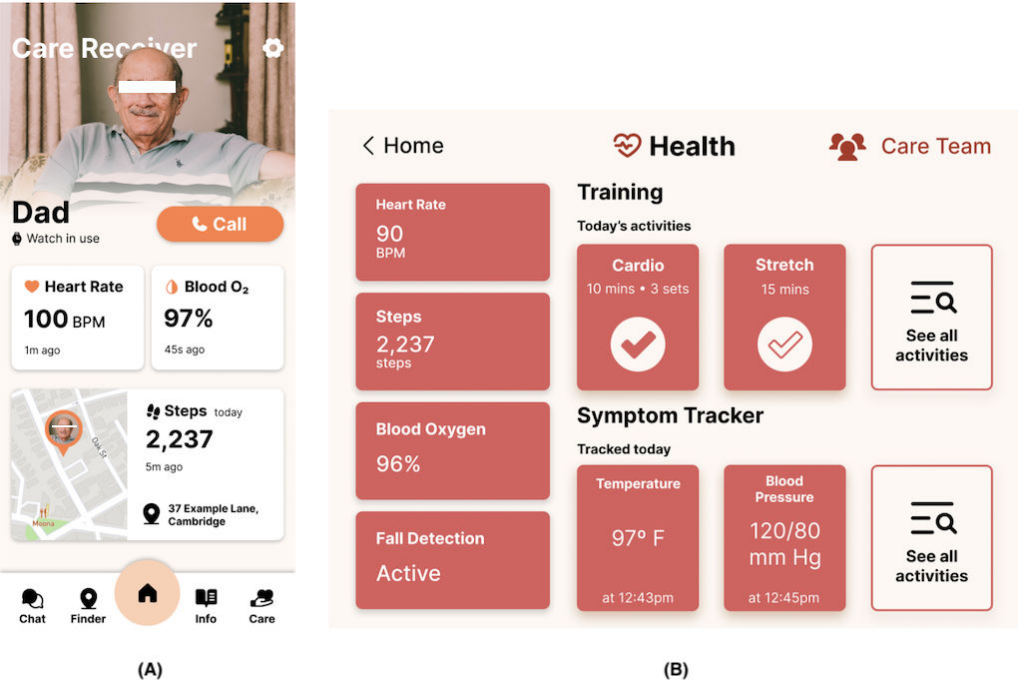
Redesigned system used for round 2: (A) Caregiver’s LifeTomorrow app; (B) Older adult’s LifeTomorrow app.

For informal caregivers, the app displays information shared by the care recipient. Informal caregivers can view vital data from their care recipients only if the care recipient chooses to share it (Figure 3). The app also includes features targeting challenges informal caregivers face, such as isolation and information scarcity [77,84]. It offers a chat feature for private conversations or a forum for public conversations, local groups for finding informal caregivers nearby, and links to resources such as blog articles and videos.

### Participants and Recruitment

Across both rounds of user studies, a total of 37 interviews were conducted with older adults, informal caregivers, and designers in the United States (see Multimedia Appendix 2). Recruitment used purposive sampling and targeted diverse roles, including informal caregivers, older adults, and designers. Older adults (aged ≥65 years [1,2]) were recruited from the MIT AgeLab Volunteer database. All older adults were in good health and were able to travel independently to the lab for study participation. Informal caregivers (nonprofessionals taking care of an older adult) came from the MIT AgeLab CareHive Panel, an open membership panel of informal caregivers who either currently provide care to an adult family member or had provided care to an adult family member in the past. Designers had at least 1 year of professional experience or relevant academic training and were recruited via snowball sampling. For the first round of user studies conducted online, participants were recruited from anywhere in the United States, whereas the second round of user studies focused on people in the Boston metropolitan area, as they were asked to come to MIT AgeLab to test the prototype in person.

In the first round of user studies, where visual mockups of the system were evaluated, 19 participants took part. There were 4 older adults (2 females and 2 males; aged 80‐92 years), 8 informal caregivers (4 females and 4 males), and 7 designers (5 females and 2 males; aged 23‐35 years).

Eighteen participants joined the second round of user studies, where an interactive prototype of the system was used. There were 9 older adults (8 females and 1 male; aged 68‐92 years), 6 informal caregivers (5 females and 1 male; aged 24‐60 years), and 3 designers (2 females and 1 male; aged 25‐34 years). Five participants from round 1 also participated in round 2. All interviews were audio and visually recorded, and the audio was transcribed for coding. Transcripts were generated automatically via Zoom or Otter.ai and reviewed by a member of the research team.

### Analysis

We conducted a qualitative thematic analysis [85] through the lens of self-determination theory [18]. Our analysis combined the interviews from both rounds of user studies to highlight themes that were dominant. The 6-phase approach by Braun and Clarke [86] guided the analysis. It involved the following steps:

1. Transcripts were reviewed, and preliminary notes were made (phase 1).
2. Inductive coding identified data relevant to the system’s impact on users’ well-being (phase 2).
3. Codes were clustered into themes, with a second researcher recoding 10% of the data from both rounds of user studies for reliability (phase 3).
4. Themes were deductively mapped to self-determination theory categories through multiple rounds of discussion, reflection, and interpretation of the content in the preliminary themes (phase 4).
5. Themes and labels were refined iteratively, resulting in 10 themes that mapped to autonomy, relatedness, or competence (phases 5 and 6), with refinement continuing throughout the writing process.

We aimed for richer interpretations of meaning rather than consensus, as guided by reflexive thematic analysis principles [87]. We contend that although many older individuals may use digital technology cautiously, they offer insightful perspectives on how technology affects society based on real-life experiences from all stages of their lives [88]. In keeping with the report by Durick et al [89], we considered older users as “specialist users,” with their specialist status deriving from their experiences in the art of living [88].

### Ethical Considerations

Participants completed a consent form prior to completing each interview and received US $75 as a gift card or via check for participation. Both rounds of user studies received exempt approval from the MIT Committee on the Use of Humans as Experimental Subjects (COUHES) with protocol numbers E-5008 and E-5079.

## Results

### Qualitative Analysis Results

**Figure 4.**
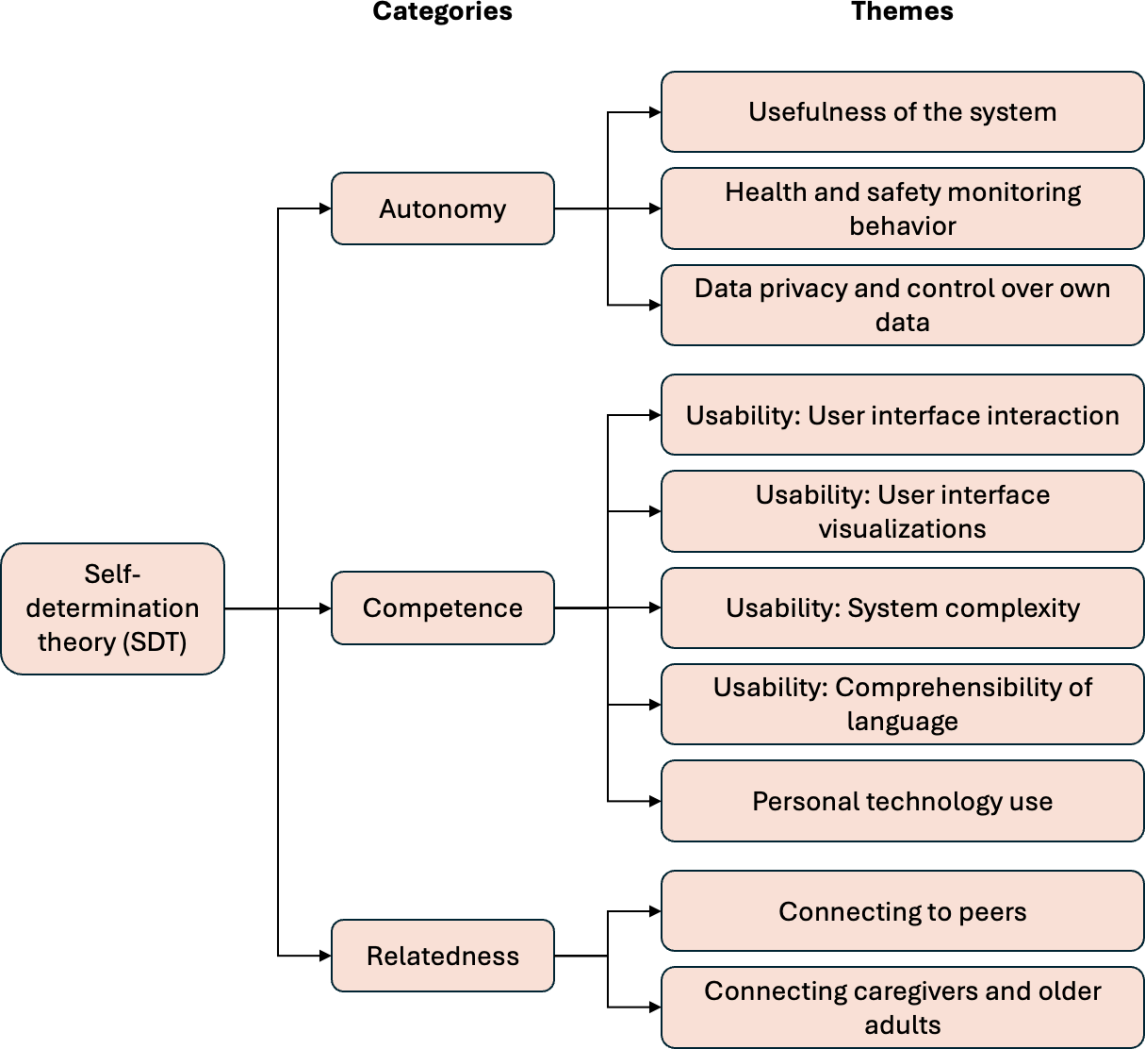
Categories and themes from the interview analysis.

Figure 4 displays the key themes that emerged from the qualitative analysis of the interviews and how they mapped to 3 categories related to self-determination.

### Autonomy

Autonomy refers to the ability to self-regulate and take ownership of one’s needs and experiences [12]. Three themes from the interviews emerged around autonomy: the usefulness or benefits expected from using the system; the value of the health and safety monitoring features to support independent living; and, for older adults in particular, the ability to exert control over sharing their data and confidence in data privacy.

#### Usefulness of the System

In the first round of user studies, most older adults reported that they found the system helpful and indicated they would use it themselves. However, 1 female individual (older adult #4) chose not to use the LifeTomorrow system due to her existing use of a paper calendar and Apple Watch. She, however, appreciated the accessibility of the system, as it allowed her to track more data and create diagrams, which could not be done with her paper calendar system.

The system’s functionalities, including health tracking, fall detection, safety sensors, and emergency alerts, were widely valued. People also appreciated having access to a range of content:

*Well, I think it is wonderful to have all the resources presented in one place. It is like an encyclopedia for an older person.*

[Older adult #1]

However, preferences for particular functions varied. Most older adults found either health tracking or safety features useful, while a minority expressed that they would derive use from both. For instance, older adult #2 desired expanded smart home sensors for added safety. Most older adults appreciated the system for fostering autonomy and managing their health data independently.

The integration of the Apple Watch into the system, rather than relying on specialized emergency devices, helped reduce stigma and increase the sense of autonomy for older adults [76,90]. Older adult #4 noted that she disliked wearing “an old lady medallion,” highlighting the watch’s appeal as a device commonly used by multiple generations.

Revisions to the system for informal caregivers after the first round of user studies, including added local groups, resource summaries, and notifications for critical vitals, were positively received in the second round of user studies. However, the divide in what features participants found useful between health tracking and smart home features remained.

Among informal caregivers, 2 groups emerged. Socially well-connected informal caregivers valued features like remote health monitoring, but they were less interested in the potential for using community and resource-sharing functionalities. In contrast, informal caregivers who felt more isolated appreciated the app’s ability to alleviate feelings of isolation through community features. One caregiver noted:

*Part of caregiving is the isolation.*

[Caregiver #5]

Regardless, informal caregivers reported that they found the app helpful overall, citing its comprehensive functionality as a significant advantage. Some concerns emerged about the validity of chat group information, but the mental health support provided by the community features was highly valued. From the feedback informal caregivers provided, tailoring resources to informal caregivers’ specific experiences and care recipients’ conditions would be essential for improving and further increasing the app’s perceived value.

#### Health and Safety Monitoring Behavior

Questions about older adults’ health and safety monitoring behaviors revealed a current reliance on simple tools like paper calendars or Apple Watches. One older adult highlighted the advantage of LifeTomorrow’s centralized data storage, making the following statement:

*I already do some of this, but it is in separate locations [paper calendar, smartwatch].*

[Older adult #4]

Safety monitoring preferences among older adults varied. The older adults in our study frequently monitored steps, heart rate, and exercise, with only older adult #8 and older adult #11 having safety monitoring besides standard smoke detectors, through a security system installed in their homes. Some informal caregivers decided to use AirTags to increase the safety of their care recipients.

Older adults appreciated the system’s affordability to provide health tracking, particularly its lack of a subscription model (older adult #4), which some participants contrasted to the service and business model of well-known Lifeline medical alert devices [91].

#### Data Privacy and Control Over One’s Own Data

Control over personal data was a central concern, emphasizing the system’s role in empowering users to decide how their data are shared. Older adults appreciated the app’s transparency and ease of managing data access. One older adult commented:

*It’s surveilling me. That’s what it’s built for, but I’m choosing to use it. [...] I have complete control over who I share [my data] with.*

[Older adult #7]

Informal caregivers valued that all shared data required care recipient consent, though some requested clearer explanations of data storage and sharing processes. The informal caregivers acknowledged the sensitivity of the data stored and shared via the app, but most older adults agreed with the informal caregivers that “there’s more value than risk” (caregiver #6). Designers noted that providing older adults with insights into how their data appear to informal caregivers can enhance trust.

### Competence

Competence is the sense that a user possesses the skills and knowledge to use the system independently and appropriately (or could acquire these relatively easily). Assessments of self-capability have been shown to be crucial for efficient work and completing moderately difficult tasks [17], and competence has been found to have a positive influence on older adults’ intentions to use smart home technologies [24].

Themes around competence emerged primarily through comments around different dimensions of ease of usability of the system, notably the interactive features and behaviors of the UI, design of its visual components, system complexity, and comprehensibility of language, and by participants’ reports of their existing experiences with technology.

For informal caregivers, accessible resources like the ones in the LifeTomorrow app addressed information gaps by providing features such as resource finders, blogs, and videos. These tools can empower informal caregivers, enhancing confidence in their abilities and reducing reliance on external support systems.

#### Usability: Interactions and UI Behaviors

Older adults were generally comfortable with touchscreens, finding the app intuitive and easy to navigate, particularly when clear descriptions (eg, “click to see history”) were added to buttons. Their confidence and competence increased as whatever they clicked “you can always go back” (older adult #9). Interacting with the iPad touchscreen was perceived as accessible and easy to learn. For instance, older adult #9 commented:

*Once I learned that you just could touch stuff and go where you want it to go. We’re off to the races.*

Informal caregivers also found the interface intuitive and suggested features like customizable home screens. Designers proposed UI improvements, such as tab bars and clearer chat functionalities, which were implemented in the second prototype, with the goals of enhancing usability and confidence for all users.

#### Usability: Visual Design

Visual accessibility and well-structured visuals were positively received. Older adult #1 emphasized this by saying:

*The presentations individually are lovely, perfect, have to be blind not to understand them.*

Moreover, older adult #5 commented:

*I love the size because I can see. […] The dividers [to organize information] are wonderful, so it is not too much that older adults do not see anything anymore.*

Older adults appreciated the block organization, high contrast, and color-coded categories, saying that these made navigation easier. Large, clear health data graphs, adapted from iOS Health, ensured quick information retrieval. Feedback led to replacing unclear icons and refining the color scheme for improved accessibility.

Informal caregivers valued the visual consistency with other iOS apps and requested indicators for data updates, which were added.

*I think that it was very clear. I feel like it feels clean. And there’s not too many things going on*.

[Caregiver #13]

Designers highlighted the importance of high-contrast colors and simple layouts. We received positive feedback on the accessibility of both apps, particularly after the second round of user studies, and it was summarized by designer #4 as “bright colors, high contrast, big text.”

#### Usability: System Complexity

The perceived complexity of an app impacts users’ confidence in using it, as apps seen as too complex can be overwhelming and hinder the psychological need for competence. LifeTomorrow’s adaptability allowed users to focus only on desired functionalities, enabling lower complexity despite its multifunctionality.

*I think I like the fact that it covers various areas. It’s not one thing onlz, it’s covering the health, your living quarters, emergencies, and so on. So, I like it. And it’s user-friendly*.

[Older adult #3]

In the second round of user studies, we found that older adults perceived the app to be intuitive and easy to navigate, as tasks, such as finding the highest heart rate of the week, were completed quickly. This low complexity fostered a high sense of competence among the older adults in our study. Older adult #4, for instance, stated how surprised she was that she could navigate around the app so intuitively. Older adult #8 described the app as “easy to look at,” referring to the amount of information displayed on every page.

*It’s very smart. It’s very clean, pleasing to the eye. It makes perfect sense. And it’s, like, maneuverability to navigate so far has been critical.*

[Older adult #9]

Informal caregivers appreciated the app’s simplicity (“it feels clean. And there’s not too many things going on” [caregiver #13]) and consistency with Apple interfaces. They emphasized the need for streamlined chat functionalities, which were redesigned for better navigation. Designers praised the system’s organization and reduced complexity in the final version.

#### Usability: Comprehensibility of Language

Clear language and descriptions improved confidence among older adults, who previously struggled with terms like “fall detection” and “care team.” Changes like replacing “hashtags” with “group chats” and “care team” with “community” reduced confusion and stigma. The second round of user studies showed significant improvements in understanding due to added explanations and user-friendly wording (eg, “training” instead of “exercise”). Overall, older adults were less likely to explore the functionality themselves if they did not know what different buttons or functions meant or led to, as older adult #6 described:

*[My] Fitbit is very dense with information I don’t understand, you know, blood glucose, […], I don’t know what that means the don’t offer explanations. So I sort of just ignore the functions. And in your system I understand what all these things are, […] zou can just glance at it and see what’s useful.*

### Relatedness

Relatedness concerns people’s connections to one another, including their social ties [12]. From the interviews, data clustered around 2 themes under relatedness: connecting to peers (among informal caregivers) and connecting informal caregivers and older adults. Technologies that foster relatedness offer the potential for reducing isolation and loneliness among informal caregivers and older adults, and in turn supporting people’s health and well-being [19,58,92].

#### Connecting to Peers

LifeTomorrow appeared to facilitate people’s relatedness to peers via health dialog and community connections, offering mutual assistance. Older adult #9, for instance, highlighted that conversations about blood oxygen levels and exercise can improve older adults’ sense of relatedness, increasing the value that LifeTomorrow provides. Informal caregivers, especially those who reported lacking a support network, valued the app’s chat feature. Caregiver #5 highlighted its importance:

*I like the idea of a community built in because part of caregiving is the isolation. I don’t necessarily want to burden people in my day-to-day life with questions. But if it’s a space that I’m opting into, the other folks are opting into. It would be really-I don’t know-It would be pleasant.*

Feeling socially connected is a fundamental human need [92], yet informal caregivers often experience isolation [93]. By incorporating features, such as a chat function, the system aimed to provide informal caregivers with opportunities for peer support, sharing both emotional and informational resources. For instance, caregiver #3 noted:

*It has just been invaluable, knowing other people who are taking care of their parents.*

Informal caregivers appreciated the ability to connect with peers virtually but also desired opportunities for in-person connections.

Older adults also expressed benefits from health-focused social interactions. Through shared knowledge, they could empower themselves and foster a sense of connection. Older adult #9 described:

*I think seniors will connect about their health. So what’s your heart rate? How many steps did you take yesterday? So the ways in which to foster positive kind of competition that I think is really a good thing that seniors get into, because they’re going to call [a friend].*

#### Connecting Informal Caregivers and Older Adults

Additionally, the system has the potential to facilitate informal caregiver–care recipient relationships by enabling older adults to control their health data and selectively share the data. Features, such as fall detection and emergency calls, may reduce informal caregivers’ anxiety about their loved ones, creating opportunities for more meaningful, nonhealth-related interactions during phone calls or visits.

Older adults valued control over sharing health data, preferring LifeTomorrow’s selective sharing approach over constant surveillance, which informal caregivers often favored. When in control of their own data, several older adults mentioned things like “I would love to data share with my primary care physicians” (older adult #8) or “I would certainly like it to tell somebody if I fallen” (older adult #2), indicating that they crave and would seek connection with their informal caregivers with regard to their health via LifeTomorrow.

While informal caregivers frequently visited or contacted their care recipients, they expressed constant worry about their safety. LifeTomorrow’s features, such as heart rate and GPS tracking, fall detection, and emergency calls, addressed these concerns, aiming to give informal caregivers more peace of mind and strengthening remote connections. For instance, when talking about the LifeTomorrow function where the care recipient can choose to share some data, caregiver #12 commented:

*What am I really going to do first thing every day, I’m really gonna go here just to check this, and I will hit this the most every single day.*

This improved sense of relatedness was pivotal for both informal caregivers and older adults, fostering trust and support in caregiving relationships.

## Discussion

### Overview

The results of this research underscore the extent to which people’s reactions to the system reflect the values they place on autonomy, competence, and relatedness, consistent with the core concepts in self-determination theory.

### Core Concepts in Self-Determination Theory Are Implicated in How People Respond to New Technologies

Technologies to support care and independent living are becoming increasingly important for an aging population as the number of older adults grows and the relative availability of informal caregivers declines. The LifeTomorrow system was designed with older users at its center, with the goal of making it easy for older adults to use it and to be in control of their data. In this research, potential users (older adults and informal caregivers) interacted with a system prototype to evaluate its design and to describe their reactions (positive and negative) to it. The responses could be grouped into 10 themes that, in turn, mapped to 3 foundational constructs of self-determination theory (see Figure 4). The results of this research underscore the extent to which people’s reactions to new technological systems reflect the values they place on autonomy, competence, and relatedness, consistent with the core concepts in self-determination theory.

From the interviews, it was apparent that older adults often strived to maintain their autonomy, avoiding safety monitoring systems that could stigmatize them as dependent or frail (eg, resistance to wearing an “old lady medallion”). The study also found that older adults are privacy-aware and prefer to have control of their own data. This aligns with prior research showing that older adults make rational, calculated decisions about information disclosures, challenging the perception that they are less privacy-conscious than younger individuals [94].

Competence, a key component of self-determination, was closely tied to the themes of usability and accessibility in the interview data. Many older adults, however, face barriers to technology proficiency, which in turn contribute to health inequities [11]. The intuitive interface design of the system prioritized simplicity, clear navigation, and accommodation for physical changes [95], and as such, the system reduced the likelihood of failed interactions, fostering user confidence. Past work has demonstrated that older adults can effectively use technology when it is designed to align with their needs and when the systems and devices are easier for people to use [13,24,88,96].

A technology-enabled solution can also foster self-determination by addressing the psychological need for relatedness in older adults and their informal caregivers. Informal caregivers described the value of knowing they were not alone, and older adults could envision how the system could serve as a point of conversation and connection with other older adults and their informal caregivers [97].

### Self-Determination Theory and a Holistic Approach to Understanding Technology Adoption

To reap the benefits that technology potentially offers to older adults and informal caregivers, older adults need to actually adopt and use new technologies. Typically, when new technologies, products, or services are designed for older adults, there is a significant focus on the characteristics of direct, easily observable interactions between the individual and the product (eg, usability, ease of use, simplicity, effectiveness, and error prevention), which have been traditionally emphasized for products where the surrounding social context is less important [98]. While the 2 main factors of perceived usefulness and ease of use in the technology acceptance model [99] have been found repeatedly to be key to user adoption of new technologies and systems, the research also points to the need for a more comprehensive consideration of the whole user experience, including the broader environment and context of use, facilitating conditions and delivery channels, related social dynamics, emotional aspects of use, and behavioral characteristics (prior experiences, beliefs, and values) [98,100,101].

While Lee and Coughlin [98] and the UTAUT2 (Unified Theory of Acceptance and Use of Technology 2) [101] specifically developed frameworks that explain how technologies may be integrated into and operated as part of a larger system, at the individual level, self-determination theory may offer a structure that can encompass many of these factors that are key for understanding technology adoption. Moreover, because of the implication of the self in self-determination theory, technology adoption and continued use may be more likely and “stickier” in cases where devices or systems arouse all 3 of the core values. For example, positive emotional responses to technologies and a perceived lack of stigma were consistent with the perspectives of Lee and Coughlin and aligned with people’s sense of autonomy. Competence, which rests on the usability and ease of use of the device or system and the ability to access support, can similarly capture some of the key findings of both Lee and Coughlin [98] and Davis [99]. Finally, relatedness can capture what Lee and Coughlin [98] describe as social support for use. Assessing the extent to which autonomy, competence, and relatedness specifically are fostered or aroused by a device or system may be key to more consistent successful adoption and prolonged use of new technologies.

Thus, self-determination theory may offer a slightly different window into understanding technology adoption and use, particularly for older users who may be less confident or comfortable using new technologies, devices, or systems. Self-determination theory provides a means to evaluate new devices or systems not simply through a checklist of accessibility needs that focus solely on system function and feel. It points beyond these to capture how people’s values, feelings, and experiences connect with the technology, which in turn may influence its use. This presents an opportunity to explore how specifically a self-determination metric might perform around gathering user evaluations or reactions to new technologies, and it opens up fruitful veins to examine whether and which different variables affect the weight or value users place on the 3 self-determination constructs (autonomy, competence, and relatedness) regarding adoption and how the weights on these values might also be affected by the purpose or function of the device.

### Future Research Possibilities

Self-determination theory offers a perspective on how core values of autonomy, competence, and relatedness shape people’s reactions to technologies. While this work demonstrates that these values are expressed through people’s evaluations of a prototype system, future work could explore more systematically how different system features relate to different core values in self-determination theory. Further, future research could explore whether different users weigh core values differently (eg, “Do older adults place more weight on the value of autonomy that a technology or system provides compared to informal caregivers?” “Do informal caregivers value competence more highly?”) and examine the extent to which these factors have an impact on user adoption of such technologies.

### Limitations

There were limitations owing to the nature of the sample: a convenience, self-selected sample for older adults and informal caregivers, and a snowball sample for designers. Limited demographic and psychographic data were collected about the user participants in this study, who are likely not representative of the population as a whole. Given their residential location and willingness to participate in the study, they may have higher levels of education and income. This user sample is also likely to be more comfortable with technologies, as many consented and participated in interviews via Zoom, and might be more likely to be among those more interested and willing to adopt and use such a system. Regardless, participants could only test the system in the interviews. Use of the system in their daily lives (in a real-world setting) might lead to different opinions and results. As our sample was relatively small with regard to older adults and informal caregivers in the system, a larger trial is strongly encouraged to obtain further insights on this topic.

### Conclusion

Technology offers the potential to support more older adults in terms of living at home independently for longer. However, to be successful, such technologies must involve systems that both older adults and informal caregivers feel confident and comfortable adopting and using. In a prototype system designed with an older adult agency at its heart, user evaluations of the system revealed that the 3 core values of self-determination theory (autonomy, competence, and relatedness) were useful for categorizing and understanding people’s reactions. Further research should explore how different kinds of system features map specifically to different core values; whether users place different weights on different values; and how autonomy, competence, and relatedness affect adoption and continued use of technologies that provide these to users.

## Supplementary material

10.2196/72579 Multimedia Appendix 1 Example study questions.

10.2196/72579 Multimedia Appendix 2 Study participant demograpics.

## Abbreviations

HCI: human-computer interaction
MIT: Massachusetts Institute of Technology
UI: user interface
