# Supplementary material for: An Accessible Multifunctional System to Support Safe and Independent Aging in Place: Iterative Development and Qualitative Analysis
Source: JMIR Aging. 2025 Oct 16;8:e72579. doi: 10.2196/72579 (PMC12530453; doi:10.2196/72579)
Supplement: Multimedia Appendix 1 [file aging-v8-e72579-s001.docx]

## Multimedia Appendix 1. Example study questions.

| Study phase | Question category | Example questions |
| --- | --- | --- |
| Both 1 and 2 | Impressions | What are your first impressions of the overall system? |
|  |  | Was there anything in particular that stood out as something that you liked / did not like? |
|  | General | What did you think about the overall usefulness of the system? |
|  |  | Is there anything obviously missing to you in the apps? Is there some kind of content that should be included but is not there right now? |
|  |  | Would you use the application yourself? |
|  |  | What are your thoughts regarding the privacy protectiveness of the application? |
|  |  | Is there anything that you would like to share regarding this topic that you have not been asked about? |
| Only study 1 | Impressions per functionality / screen on the mockups | What did you think about the usefulness of the home tab on the app? |
|  |  | What features did you like the most in the home tab? |
|  |  | What features did you miss in a home tab? |
| Only study 2 | Interaction with the prototype – with only minor narrative intro to the system, the participants were asked to interact with it. | Can you please find your (for older adults) / your care recipients (for caregivers) heart rate? |
|  |  | Caregivers: Can you please find a lawyer that is specialized in caregiving with the system? |
|  |  | Older adults: Can you please share your heart rate with the fictional caregiver, Joe Canton? |
|  |  | Older adults: Please tell me on which day you had the highest step count. |
