# Supplementary material for: An Accessible Multifunctional System to Support Safe and Independent Aging in Place: Iterative Development and Qualitative Analysis
Source: JMIR Aging. 2025 Oct 16;8:e72579. doi: 10.2196/72579 (PMC12530453; doi:10.2196/72579)
Supplement: Multimedia Appendix 2 [file aging-v8-e72579-s002.docx]

## Multimedia Appendix 2. Study participant demographics.

| Interview Number | Study Number | Participant Group | ID | Age | Biological sex | Participated in both studies |
| --- | --- | --- | --- | --- | --- | --- |
| 1 | 1 | Older adult | OA1 | 88 | Male | No |
| 2 | 1 | Older adult | OA2 | 90 | Male | Yes |
| 3 | 1 | Older adult | OA3 | 80 | Female | No |
| 4 | 1 | Older adult | OA4 | 92 | Female | Yes |
| 5 | 1 | Caregiver | CG1 | n/a | Female | No |
| 6 | 1 | Caregiver | CG2 | n/a | Female | No |
| 7 | 1 | Caregiver | CG3 | n/a | Male | Yes |
| 8 | 1 | Caregiver | CG4 | n/a | Female | No |
| 9 | 1 | Caregiver | CG5 | n/a | Male | No |
| 10 | 1 | Caregiver | CG6 | n/a | Male | No |
| 11 | 1 | Caregiver | CG7 | n/a | Female | No |
| 12 | 1 | Caregiver | CG8 | n/a | Male | No |
| 13 | 1 | Designer | DS1 | 35 | Male | No |
| 14 | 1 | Designer | DS2 | 30 | Female | No |
| 15 | 1 | Designer | DS3 | 32 | Male | Yes |
| 16 | 1 | Designer | DS4 | 25 | Female | Yes |
| 17 | 1 | Designer | DS5 | 31 | Female | No |
| 18 | 1 | Designer | DS6 | 23 | Female | No |
| 19 | 1 | Designer | DS7 | 29 | Female | No |
| 20 | 2 | Older adult | OA5 | 69 | Female | No |
| 21 | 2 | Older adult | OA6 | 68 | Female | No |
| 22 | 2 | Older adult | OA7 | 74 | Female | No |
| 23 | 2 | Older adult | OA8 | 72 | Female | No |
| 24 | 2 | Older adult | OA2 | 90 | Male | Yes |
| 25 | 2 | Older adult | OA9 | 69 | Female | No |
| 26 | 2 | Older adult | OA4 | 92 | Female | Yes |
| 27 | 2 | Older adult | OA10 | 70 | Female | No |
| 28 | 2 | Older adult | OA11 | 72 | Female | No |
| 29 | 2 | Caregiver | CG9 | 51 | Female | No |
| 30 | 2 | Caregiver | CG10 | 24 | Female | No |
| 31 | 2 | Caregiver | CG3 | 55 | Male | Yes |
| 32 | 2 | Caregiver | CG11 | 60 | Female | No |
| 33 | 2 | Caregiver | CG12 | 52 | Female | No |
| 34 | 2 | Caregiver | CG13 | 47 | Female | No |
| 35 | 2 | Designer | DS8 | 34 | Female | No |
| 36 | 2 | Designer | DS3 | 32 | Male | Yes |
| 37 | 2 | Designer | DS4 | 25 | Female | Yes |
